# Supplementary material for: Object representations in the human brain reflect the co-occurrence statistics of vision and language
Source: Nat Commun. 2021 Jul 2;12:4081. doi: 10.1038/s41467-021-24368-2 (PMC8253839; doi:10.1038/s41467-021-24368-2)
Supplement: Supplementary file 1 — Supplementary Information [file 41467_2021_24368_MOESM1_ESM.pdf]

SUPPLEMENTARY INFORMATION

Supplementary figures for:

Object representations in the human brain reflect the co-occurrence statistics of vision and language

Michael F. Bonner and Russell A. Epstein

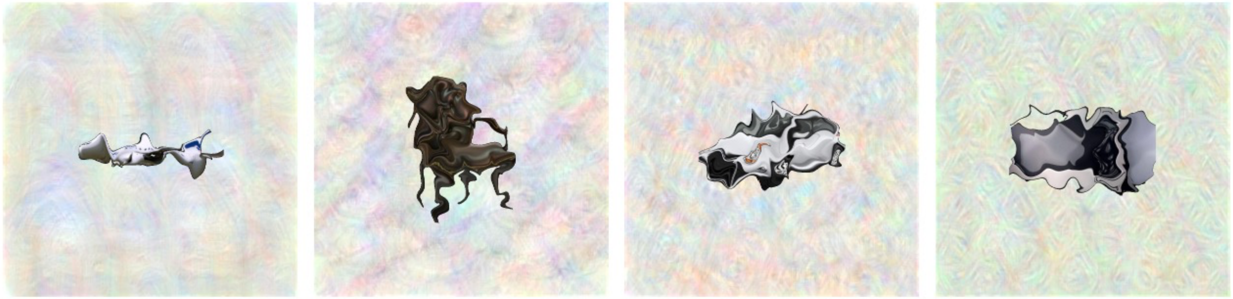

Supplementary Figure 1. Warped object stimuli. In the fMRI experiment, subjects were asked to press a button whenever they saw an image of a warped object. These warped objects were created by applying a diffeomorphic warping algorithm to the images in our stimulus set. This figure shows examples of warped images for four different categories of objects.

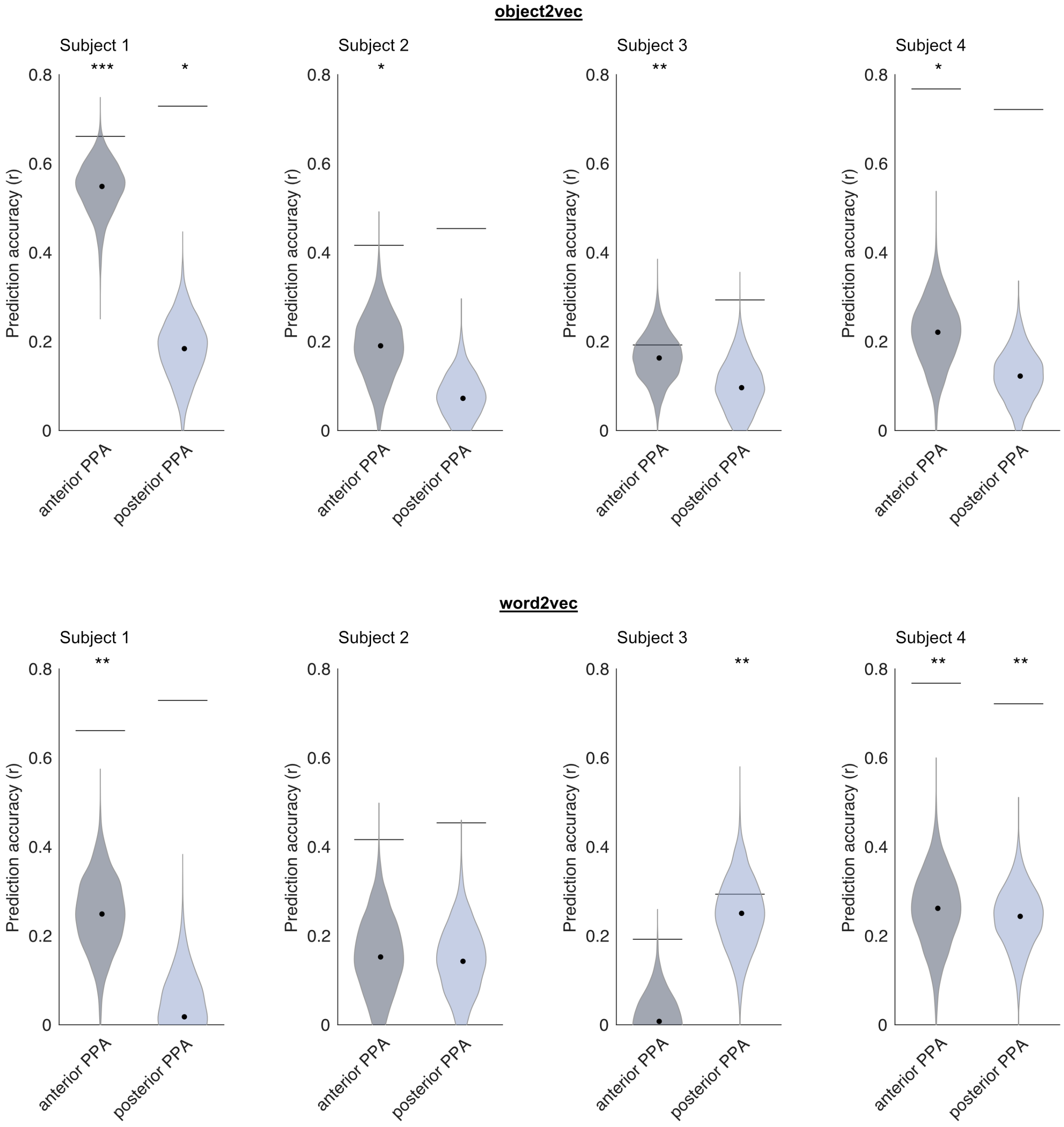

Supplementary Figure 2. Voxelwise encoding models of object context in PPA for single subjects. This plot shows the average prediction accuracies for encoding models in voxels from the anterior third and posterior third of the PPA using either image-based object2vec representations as regressors or language-based word2vec representations. The violin plots show the mean prediction accuracies (central black dots) and bootstrap standard deviations. The gray lines above each violin plot indicate the average voxelwise split-half reliability of the fMRI responses in each ROI. *p<0.05, **p<0.01, ***p<0.001, uncorrected, one-sided permutation test. Exact p-values for object2vec: Subject 1 anterior PPA p-value=2.0e-04; Subject 1 posterior PPA p-value=2.2e-02; Subject 2 anterior PPA p-value=2.2e-02; Subject 2 posterior PPA p-value=1.9e-01; Subject 3 anterior PPA p-value=1.0e-02; Subject 3

posterior PPA p-value=1.2e-01; Subject 4 anterior PPA p-value=1.6e-02; Subject 4 posterior PPA p-value=6.1e-02. Exact p-values for word2vec: Subject 1 anterior PPA p-value=5.4e-03; Subject 1 posterior PPA p-value=4.3e-01; Subject 2 anterior PPA p-value=7.3e-02; Subject 2 posterior PPA p-value=5.1e-02; Subject 3 anterior PPA p-value=4.6e-01; Subject 3 posterior PPA p-value=2.4e-03; Subject 4 anterior PPA p-value=3.2e-03; Subject 4 posterior PPA p-value=1.8e-03. Source data are provided as a Source Data file.

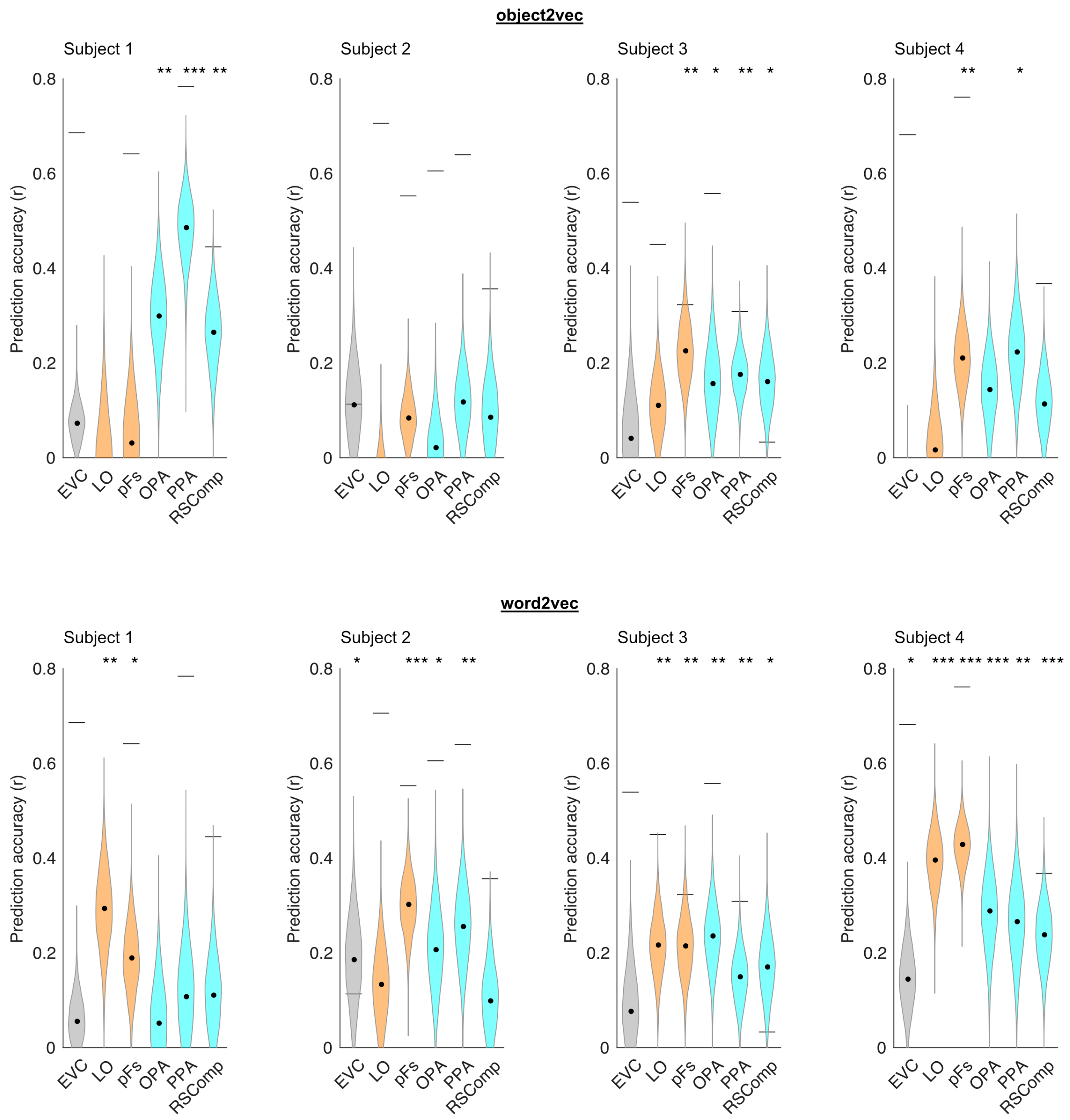

Supplementary Figure 3. Voxelwise encoding models of object context in functionally defined ROIs for single subjects. This plot shows the average prediction accuracies for encoding models in voxels from multiple regions of interest using either image-based object2vec representations as regressors or language-based word2vec representations. Object-selective ROIs are plotted in orange and scene-selective ROIs are plotted in cyan. The violin plots show the mean prediction accuracies (central black dots) and bootstrap standard deviations. The gray lines above each violin plot indicate the average voxelwise split-half reliability of the fMRI responses in each ROI. EVC = early visual cortex, LO = lateral occipital, pFs = posterior fusiform, OPA = occipital place area, PPA = parahippocampal place area, RSComp = retrosplenial complex. *p<0.05, **p<0.01, ***p<0.001, uncorrected, one-sided permutation test. Exact p-values for object2vec: Subject 1 EVC p-value=1.1e-01; Subject 1 LO p-value=5.3e-01; Subject 1 pFs p-value=3.8e-01; Subject 1 OPA p-value=2.6e-03; Subject 1 PPA p-value=2.0e-04; Subject 1 RSComp p-value=4.6e-03;

Subject 2 EVC p-value=1.4e-01; Subject 2 LO p-value=8.4e-01; Subject 2 pFs p-value=1.3e-01; Subject 2 OPA p-value=4.0e-01; Subject 2 PPA p-value=1.0e-01; Subject 2 RSComp p-value=1.8e-01; Subject 3 EVC p-value=3.3e-01; Subject 3 LO p-value=1.0e-01; Subject 3 pFs p-value=2.4e-03; Subject 3 OPA p-value=4.5e-02; Subject 3 PPA p-value=5.8e-03; Subject 3 RSComp p-value=2.9e-02; Subject 4 EVC p-value=9.9e-01; Subject 4 LO p-value=4.2e-01; Subject 4 pFs p-value=1.4e-03; Subject 4 OPA p-value=6.9e-02; Subject 4 PPA p-value=2.3e-02; Subject 4 RSComp p-value=8.0e-02. Exact p-values for word2vec: Subject 1 EVC p-value=1.9e-01; Subject 1 LO p-value=1.4e-03; Subject 1 pFs p-value=1.9e-02; Subject 1 OPA p-value=3.2e-01; Subject 1 PPA p-value=1.5e-01; Subject 1 RSComp p-value=1.2e-01; Subject 2 EVC p-value=2.1e-02; Subject 2 LO p-value=6.2e-02; Subject 2 pFs p-value=2.0e-04; Subject 2 OPA p-value=1.6e-02; Subject 2 PPA p-value=4.6e-03; Subject 2 RSComp p-value=1.2e-01; Subject 3 EVC p-value=2.0e-01; Subject 3 LO p-value=3.2e-03; Subject 3 pFs p-value=1.0e-03; Subject 3 OPA p-value=1.6e-03; Subject 3 PPA p-value=5.6e-03; Subject 3 RSComp p-value=1.9e-02; Subject 4 EVC p-value=2.4e-02; Subject 4 LO p-value=2.0e-04; Subject 4 pFs p-value=2.0e-04; Subject 4 OPA p-value=1.6e-03; Subject 4 PPA p-value=4.8e-03; Subject 4 RSComp p-value=6.0e-04. Source data are provided as a Source Data file.

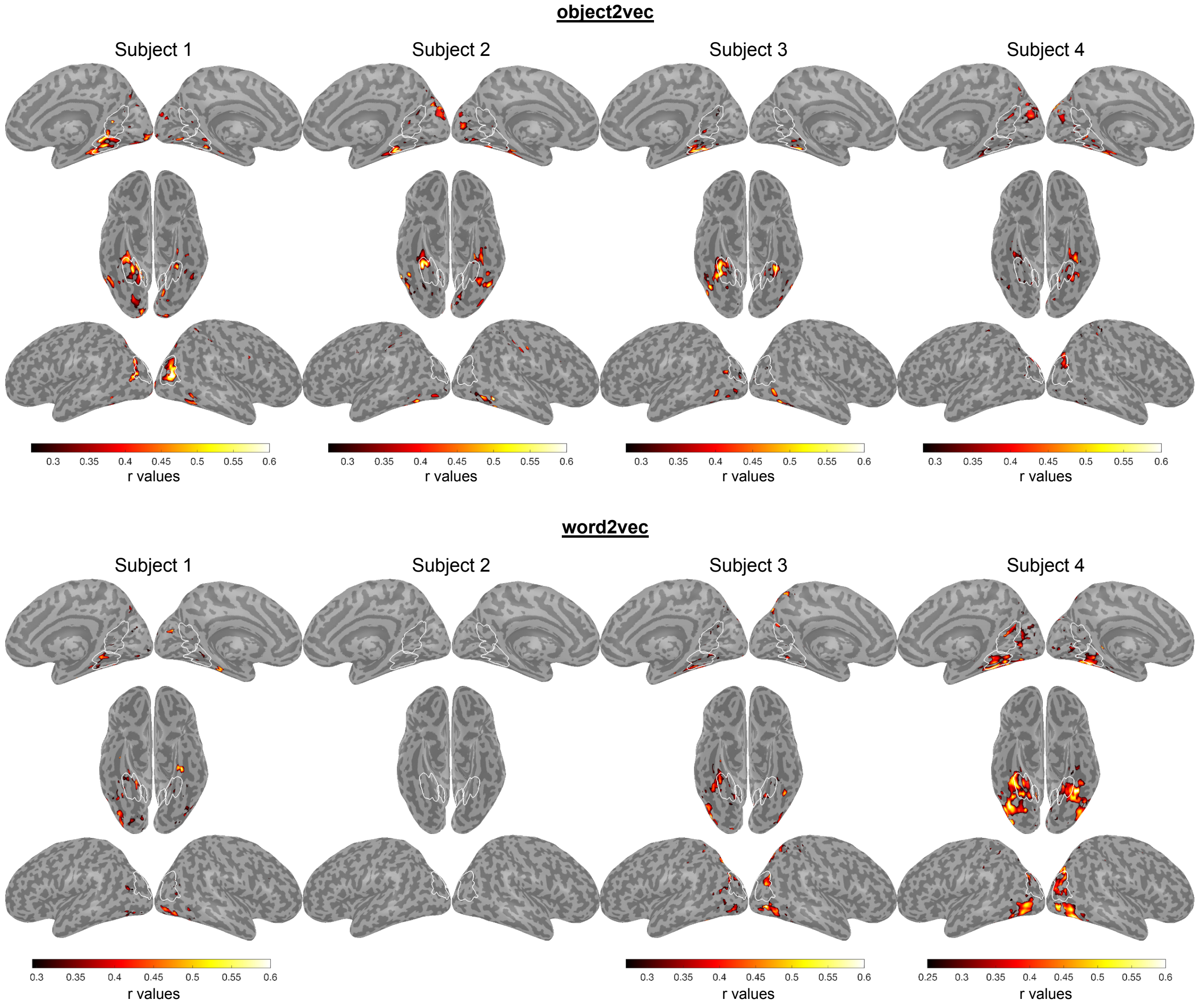

Supplementary Figure 4. Whole-brain voxelwise encoding models of object context for single subjects. Voxelwise encoding models were estimated and assessed using the 9-fold cross-validation procedure described in Figure 3. These analyses were performed for all voxels with split-half reliability scores greater than or equal to r = 0.1841, which corresponds to a one-sided, uncorrected p-value of 0.05 (see split-half reliability mask in Fig. S8). Encoding model accuracy scores are plotted for voxels that show significant effects ($p<0.05$, FDR-corrected, one-sided permutation test). The top panel shows prediction accuracy for the image-based object2vec encoding model, and the bottom panel shows prediction accuracy for the language-based word2vec encoding model. ROI parcels are shown for scene-selective ROIs, as in Fig.6.

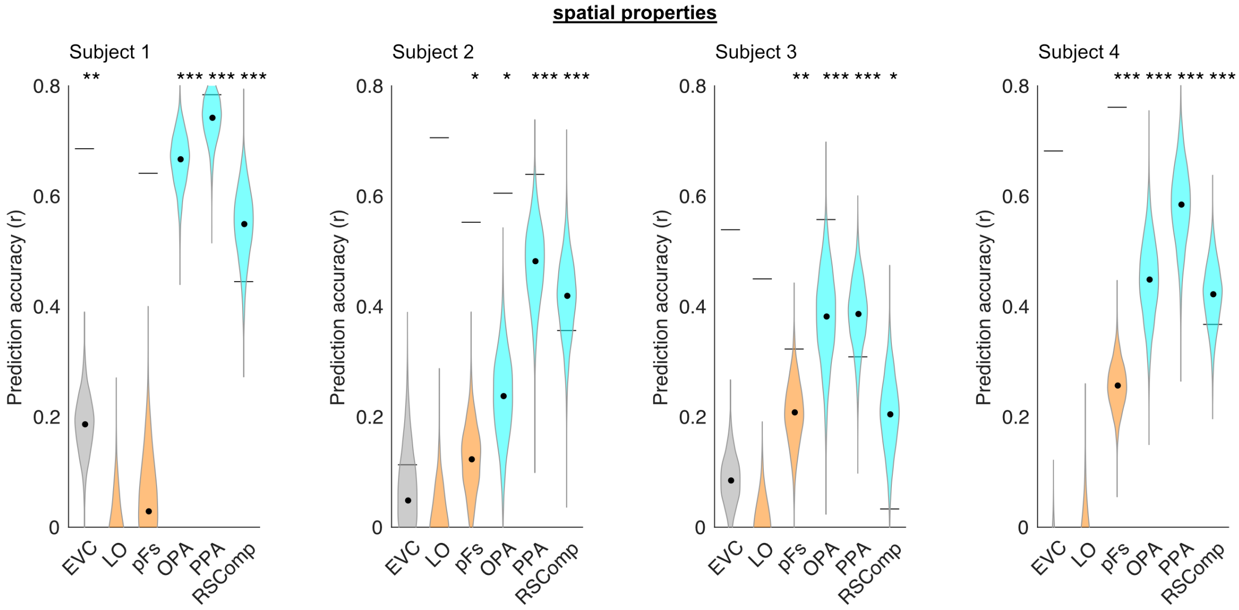

Supplementary Figure 5. Voxelwise encoding models of object spatial properties in functionally defined ROIs for single subjects. This plot shows the average prediction accuracies for encoding models in voxels from multiple regions of interest using spatial-property ratings as regressors (i.e., real-world size and spatial stability). Object-selective ROIs are plotted in orange and scene-selective ROIs are plotted in cyan. The violin plots show the mean prediction accuracies (central black dots) and bootstrap standard deviations. The gray lines above each violin plot indicate the average voxelwise split-half reliability of the fMRI responses in each ROI. EVC = early visual cortex, LO = lateral occipital, pFs = posterior fusiform, OPA = occipital place area, PPA = parahippocampal place area, RSComp = retrosplenial complex. *p<0.05, ***p<0.01, ***p<0.001, uncorrected, one-sided permutation test. Exact p-values: Subject 1 EVC p-value=1.8e-03; Subject 1 LO p-value=7.5e-01; Subject 1 pFs p-value=3.9e-01; Subject 1 OPA p-value=2.0e-04; Subject 1 PPA p-value=2.0e-04; Subject 1 RSComp p-value=2.0e-04; Subject 2 EVC p-value=3.2e-01; Subject 2 LO p-value=5.4e-01; Subject 2 pFs p-value=4.5e-02; Subject 2 OPA p-value=1.2e-02; Subject 2 PPA p-value=2.0e-04; Subject 2 RSComp p-value=2.0e-04; Subject 3 EVC p-value=8.3e-02; Subject 3 LO p-value=5.8e-01; Subject 3 pFs p-value=7.6e-03; Subject 3 OPA p-value=2.0e-04; Subject 3 PPA p-value=2.0e-04; Subject 3 RSComp p-value=1.4e-02; Subject 4 EVC p-value=9.6e-01; Subject 4 LO p-value=8.9e-01; Subject 4 pFs p-value=2.0e-04; Subject 4 OPA p-value=2.0e-04; Subject 4 PPA p-value=2.0e-04; Subject 4 RSComp p-value=2.0e-04. Source data are provided as a Source Data file.

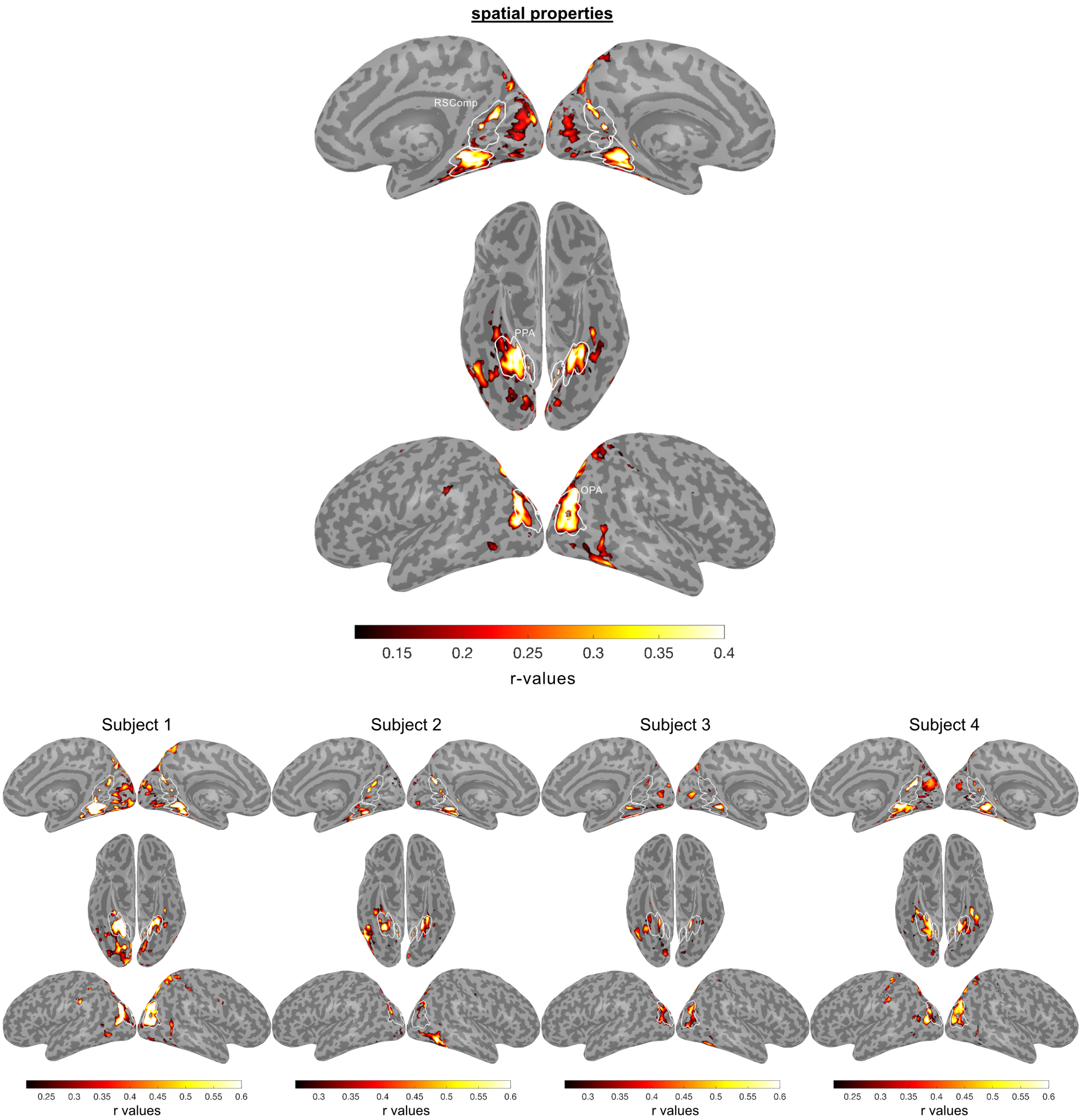

Supplementary Figure 6. Whole-brain voxelwise encoding models of object spatial properties. Voxelwise encoding models were estimated and assessed using the 9-fold cross-validation procedure described in Figure 3. These analyses were performed for all voxels with split-half reliability scores greater than or equal to r = 0.1841, which corresponds to a one-sided, uncorrected p-value of 0.05 (see split-half reliability mask in Fig. S8). Encoding model accuracy scores are plotted for voxels that show significant effects ($p<0.05$, FDR-corrected, one-sided permutation test). The top panel shows the group-average result, and the bottom panel shows single-subject results. ROI parcels are shown for scene-selective ROIs. OPA = occipital place area, PPA = parahippocampal place area, RSComp = retrosplenial complex.

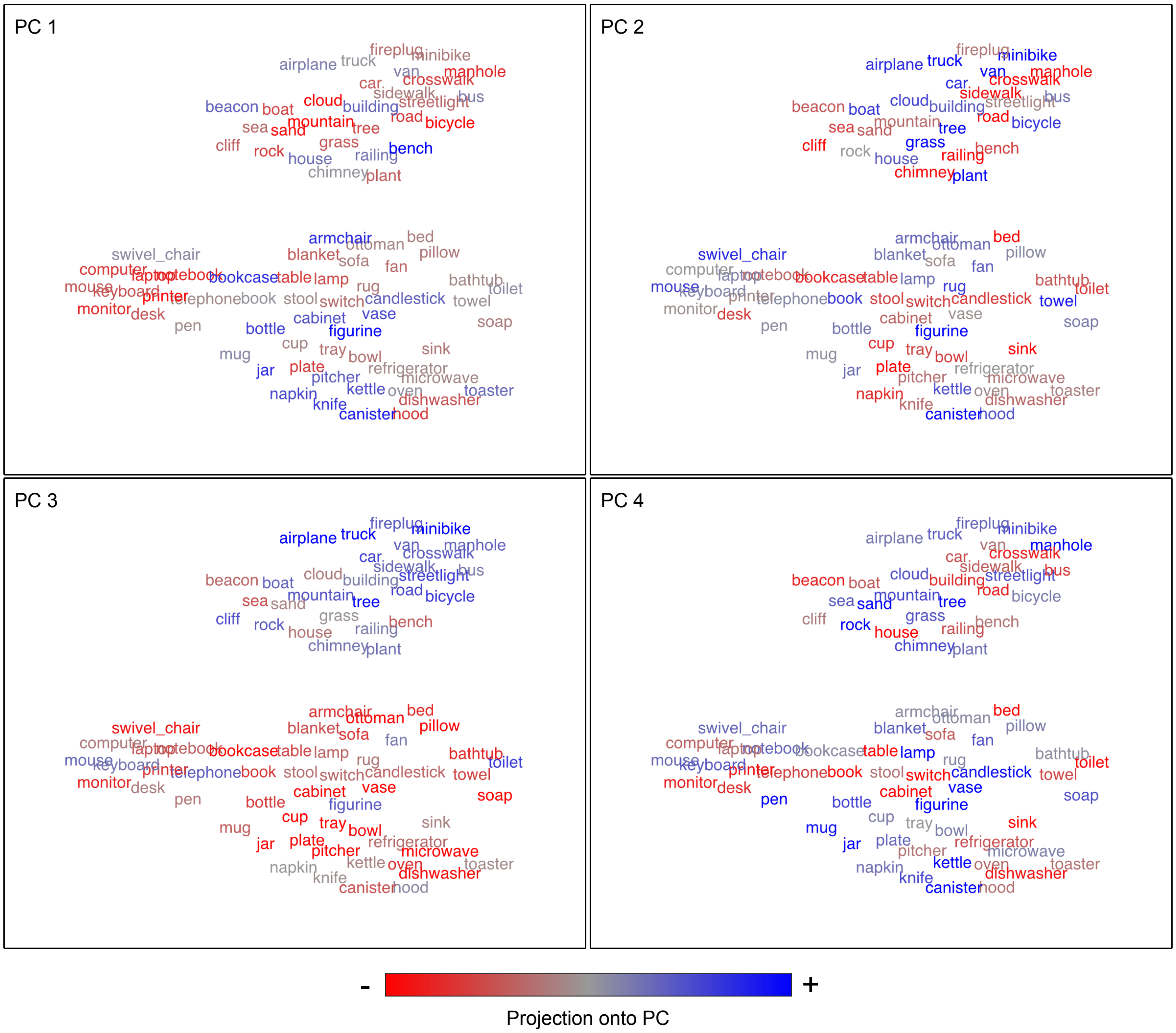

Supplementary Figure 7. Principal components of voxel tuning for language-based object context. Principal components analysis was used to examine variance in encoding-model regression weights across voxels. This plot illustrates the first four principal components (PCs) of the regression weights for the language-based word2vec encoding model, using all voxels with significant prediction accuracies. The 81 object categories from the fMRI experiment were projected onto each PC (as indicated by the color coding from blue to red). The spatial arrangement of the object categories is the same as the tSNE plot in Figure 1. Source data are provided as a Source Data file.

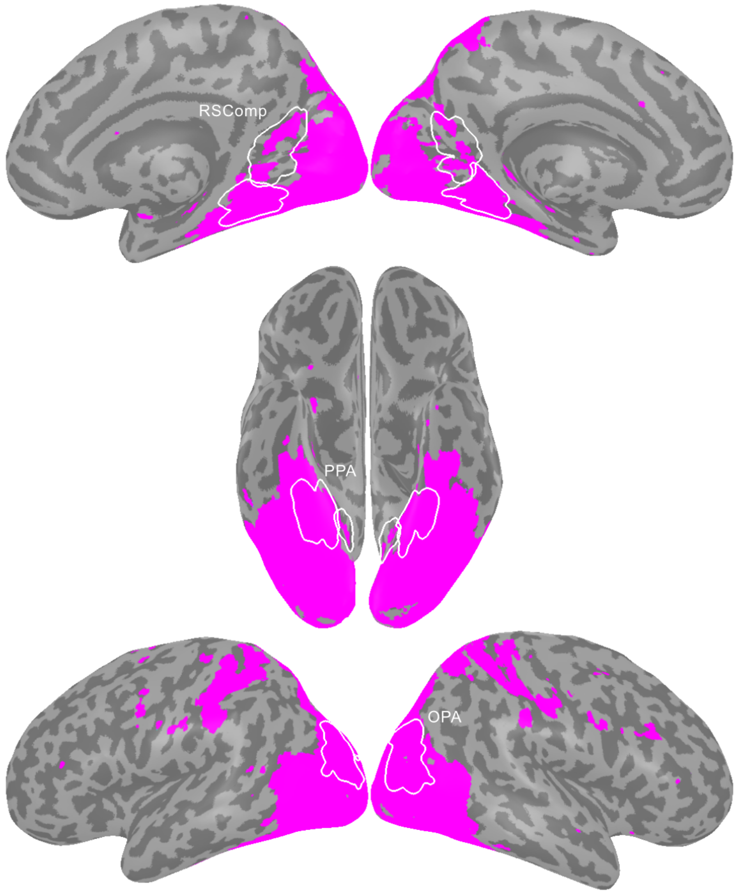

Supplementary Figure 8. Split-half reliability mask. Encoding model analyses were performed for all voxels with split-half reliability scores greater than or equal to r = 0.1841, which corresponds to a one-sided, uncorrected p-value of 0.05. For each voxel, split-half reliability was calculated as the Pearson correlation between the mean responses to the 81 object categories in odd and even runs. The purple regions on this cortical surface rendering indicate voxels that surpassed the reliability threshold. ROI parcels are shown for scene-selective ROIs. PPA = parahippocampal place area, OPA = occipital place area, RSComp = retrosplenial complex.

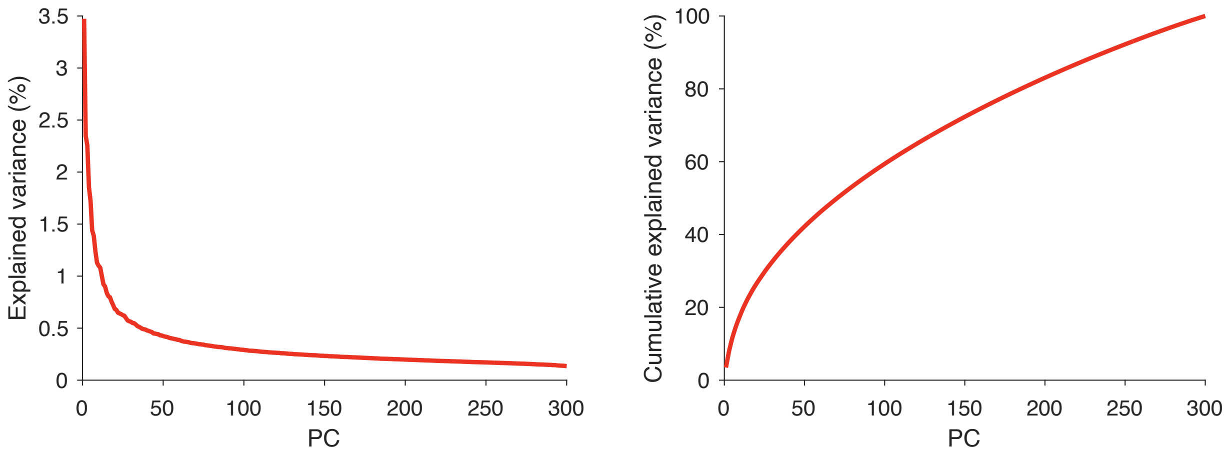

Supplementary Figure 9. Explained variance of word2vec principal components. Explained variance of principal components after applying principal components analysis to the entire word2vec dataset (207,147 words). The left panel shows the explained variance for each principal component and the right panel shows the cumulative explained variance of these components. PC = principal component. Source data are provided as a Source Data file.

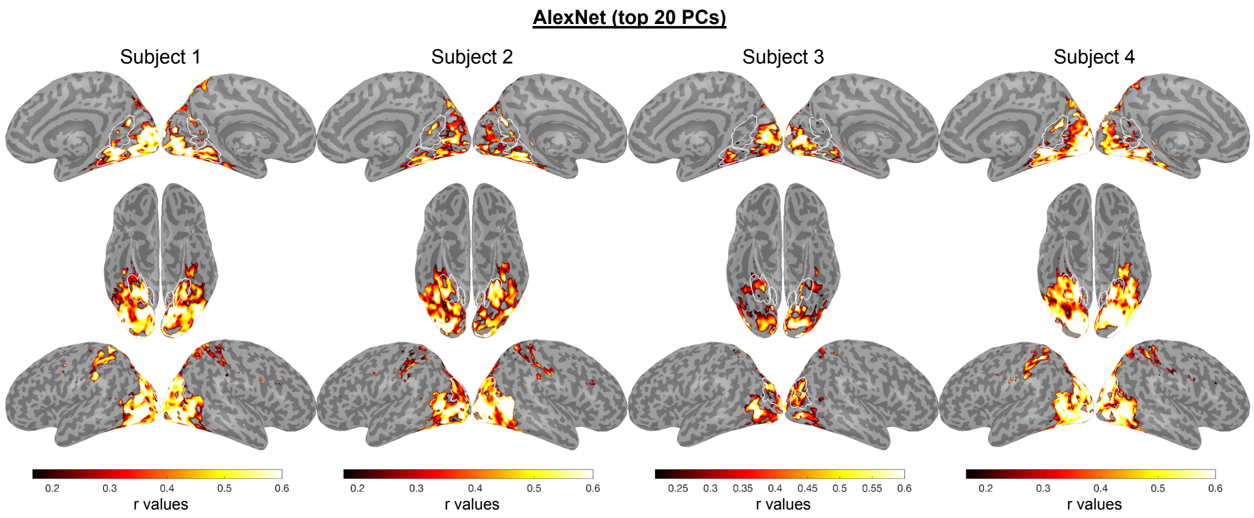

Supplementary Figure 10. Whole-brain voxelwise encoding models of AlexNet PCs for single subjects. Voxelwise encoding models were estimated and assessed using the 9-fold cross-validation procedure described in Figure 3. These analyses were performed for all voxels with split-half reliability scores greater than or equal to r = 0.1841, which corresponds to a one-sided, uncorrected p-value of 0.05 (see split-half reliability mask in Fig. S8). Encoding model accuracy scores are plotted for voxels that show significant effects ($p<0.05$, FDR-corrected, one-sided permutation test). ROI parcels are shown for scene-selective ROIs, as in Fig.6. PCs = principal components.

Supplementary Table 1. Object categories used in the fMRI experiment. This table shows all 81 object categories from the fMRI experiment, along with their associated labels in the ADE20K dataset.

Category	Label 1	Label 2	Label 3	Label 4
airplane	airplane, aeroplane, plane	seaplane, hydroplane		
armchair	armchair	rocking chair, rocker		
bathtub	bathtub, bathing tub, bath, tub	shower		
beacon	beacon, lighthouse, beacon light, pharos			
bed	bed			
bench	bench			
bicycle	bicycle, bike, wheel, cycle			
blanket	blanket, cover	blankets		
boat	boat	sailboat, sailing boat		
book	book			
bookcase	bookcase			
bottle	bottle			
bowl	bowl	bowls		
building	building, edifice	skyscraper	skyscrapers	
bus	bus, autobus, coach, charabanc, double-decker, jitney, motorbus, motorcoach, omnibus, passenger vehicle			
cabinet	cabinet			
candlestick	candlestick, candle holder			
canister	canister, cannister, tin			
car	car, auto, automobile, machine, motorcar			
chimney	chimney			
cliff	cliff, drop, drop-off			
cloud	cloud	clouds		
computer	computer, computing machine, computing device, data processor, electronic computer, information processing system			
crosswalk	crosswalk			
cup	cup	cups	glass, drinking glass	
desk	desk			
dishwasher	dishwasher, dish washer, dishwashing machine			
fan	fan			
figurine	figurine, statuette			
fireplug	fireplug, fire hydrant, plug			
grass	grass			
hood	hood, exhaust hood			
house	house			

jar	jar			
kettle	kettle, boiler	teapot		
keyboard	keyboard			
knife	knife	kitchen utensils	utensils	
lamp	lamp			
laptop	laptop, laptop computer			
manhole	manhole			
microwave	microwave, microwave oven	microwave		
minibike	minibike, motorbike			
monitor	monitor			
mountain	mountain, mount			
mouse	mouse	mouse, computer mouse		
mug	mug			
napkin	napkin, table napkin, serviette			
notebook	notebook	notepad		
ottoman	ottoman, pouf, pouffe, puff, hassock			
oven	oven	stove, kitchen stove, range, kitchen range, cooking stove	stove	
pen	pen	pens		
pillow	pillow			
pitcher	pitcher, ewer			
plant	plant	plant, flora, plant life		
plate	plate			
printer	printer			
railing	railing	railing, rail		
refrigerator	refrigerator, icebox			
road	road	road, route	roads	
rock	rock, stone	rock	rocks	pebbles
rug	rug, carpet, carpeting			
sand	sand			
sea	sea			
sidewalk	sidewalk	sidewalk, pavement		
sink	sink			
soap	soap	soap bar		
sofa	sofa, couch, lounge			
stool	stool	stools		
streetlight	streetlight, street lamp	street lights		

switch	switch, electric switch, electrical switch	light switch	switch	switches
swivel_chair	swivel chair			
table	table	tables		
telephone	telephone, phone, telephone set	telephone		
toaster	toaster	toaster oven		
toilet	toilet, can, commode, crapper, pot, potty, stool, throne			
towel	towel			
tray	tray			
tree	tree	trees		
truck	truck, motortruck			
van	van			
vase	vase	vases		
